# Integrated model simulates bigger, sweeter tomatoes under changing climate under reduced nitrogen and water input

**DOI:** 10.1093/hr/uhad045

**Published:** 2023-03-13

**Authors:** Huiping Zhou, Shaozhong Kang, Michel Génard, Gilles Vercambre, Jinliang Chen

**Affiliations:** Center for Agricultural Water Research in China, China Agricultural University, Beijing 100083, China; National Field Scientific Observation and Research Station on Efficient Water Use of Oasis Agriculture in Wuwei of Gansu Province, Wuwei 733009, China; Center for Agricultural Water Research in China, China Agricultural University, Beijing 100083, China; National Field Scientific Observation and Research Station on Efficient Water Use of Oasis Agriculture in Wuwei of Gansu Province, Wuwei 733009, China; INRAE, UR 1115 Plantes et Systèmes de Culture Horticoles, Avignon Cedex 9 F-84914, France; INRAE, UR 1115 Plantes et Systèmes de Culture Horticoles, Avignon Cedex 9 F-84914, France; Center for Agricultural Water Research in China, China Agricultural University, Beijing 100083, China; National Field Scientific Observation and Research Station on Efficient Water Use of Oasis Agriculture in Wuwei of Gansu Province, Wuwei 733009, China

## Abstract

When simulating the response of fruit growth and quality to environmental factors and cultivation practices, the interactions between the mother plant and fruit need to be considered as a whole system. Here, we developed the integrative Tomato plant and fruit Growth and Fruit Sugar metabolism (TGFS) model by coupling equations describing the biophysical processes of leaf gas exchange, water transport, carbon allocation, organ growth and fruit sugar metabolism. The model also accounts for effects of soil nitrogen and atmospheric CO_2_ concentration on gaseous exchange of water and carbon by the leaf. With different nitrogen and water input values, TGFS performed well at simulating the dry mass of the tomato leaf, stem, root, and fruit, and the concentrations of soluble sugar and starch in fruit. TGFS simulations showed that increasing air temperature and CO_2_ concentration has positive effects on fruit growth, but not on sugar concentrations. Further model-based analyses of cultivation scenarios suggest that, in the context of climate change, decreasing N by 15%–25% and decreasing irrigation by 10%–20% relative to current levels would increase tomato fresh weight by 27.8%–36.4% while increasing soluble sugar concentration by up to 10%. TGFS provides a promising tool to optimise N and water inputs for sustainable high-quality tomatoes.

## Introduction

Sustainable development of tomato (*Solanum lycopersicum*) production aims to ensure sufficient yields of good-quality fruit. Reconciling productivity, quality, and sustainability in the context of global climate change is a critical issue facing agriculture, especially for field-grown tomatoes the environment of which is less controlled. Marked rises in air temperatures and CO_2_ concentrations will affect stomatal conductance [1], photosynthesis [2], plant growth [3], fruit development [4], and fruit sugar metabolism [5]. Modelling approaches have been widely used to forecast the impacts of climate change on future agricultural productivity and assess options for local stakeholders [6]. For example, considering water shortages and fertiliser pollution [7], farmers need to follow scientific guidance to implement suitable and sustainable agricultural practices. Currently, however, climate-change impact research mainly focuses on crop growth [8–10]. Few studies consider fruit quality or comprehensively link plant growth and fruit quality.

The fruit growth pattern and composition of tomato are complex traits as they result from many processes that show large genotype, environment, and agronomic management interactions [11]. The effects of several meteorological and agronomic factors on the growth and fruit quality of tomato have been evaluated, such as radiation [12], temperature [4], the difference between the saturated vapour pressure and the actual vapour pressure (VPD) [13], irrigation, nitrogen (N) application [14], CO_2_ fertilization and in combination with reduced irrigation regimes [15, 16]. However, the effect of CO_2_ fertilization in combination with temperature, irrigation and N conditions on tomato growth and fruit quality is far less studied.

The expansive growth and sugar concentration of fruit, major quality criteria, are mainly determined by the transport and accumulation of water and carbon from the mother plant [17]. The water and carbon statuses of leaf (resp. fruit) affect the source (resp. sink) activity, thus influencing the overall source-sink balance of the whole plant-fruit system [18]. Therefore, to better understand the response of fruit growth and quality to changes in environmental factors and agronomic management, it is necessary to consider the water and carbon relations at the whole-plant level.

Process-based modelling is a powerful approach to deal with the complexity of biological systems of plants, their fruit, and the relationships between them [11, 19]. A virtual fruit model was developed to describe the water and carbon fluxes occurring during peach fruit expansive growth [20, 21]. The model has since been successfully adapted to simulate fruit growth and sugar metabolism in tomato [17, 22]. Model-assisted analyses have been conducted to assess the sensitivity of various physiological processes to water deficits and the consequence on vegetative growth and fruit quality [23], to compare sugar accumulation across different fruit species [24], and to improve the simulation of carbohydrate accumulation in tomato fruit by considering water status [25]. Fruit simulation studies have also been scaled up from the organ to the whole-plant level. For example, equations describing water transport and nutrient fluxes involved in fruit growth have been connected to the growth model to depict the coordinated distribution of water and carbon among different organs of the whole plant [26]. Such a model was applied to capture how carbon is assimilated and allocated to the main compartments and how these processes vary depending on environmental conditions, including water stress [23, 27], changes in leaf-to-fruit ratios [28], and agronomic practices such as pruning and fruit thinning [29].

Tomato is an indeterminate crop, that is, vegetative and reproductive growth is inseparable [11]. Dynamic simulation of growth of both developmental phases is challenging because it requires the integration of information on the interactions between source-sink activities within the plant, cultivation practices, and environmental factors [17, 28, 29]. There are currently several limitations. For example, most of the existing models assume that plant water status fluctuates within a given range [26, 30] and few of them consider the influence of N application and atmospheric CO_2_ concentrations on plant growth, fruit metabolism or fruit quality, especially when excess N is applied or as the climate changes [7, 31, 32].

Our first aim was to develop a process-based model for tomato to simulate the dynamic growth of the plant and its fruit, while following fruit sugar metabolism at a whole-plant level (TGFS model), and representing the impacts of N and CO_2_ on leaf gas exchange. The second aim was to predict how climate change will influence tomato organ growth and fruit sugar composition, as a way to investigate management strategies for sustainable tomato production in the future.

## Results

### Calibration and validation of the integrative model

**Figure 1:**
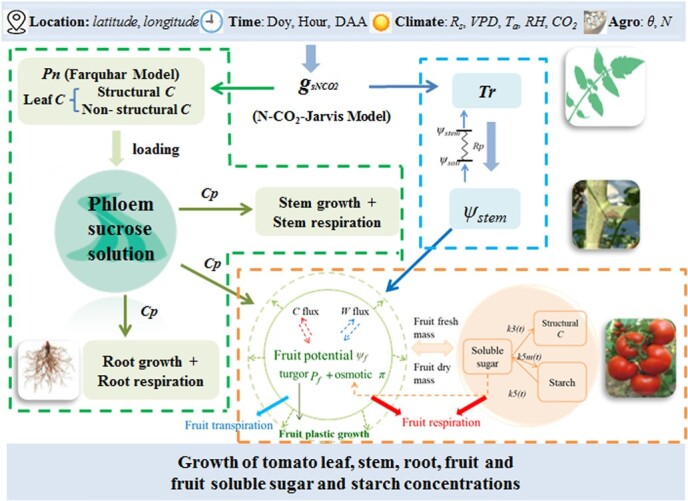
Schematic diagram of the integrated Tomato plant and fruit Growth and Fruit Sugar metabolism (TGFS) model. The inputs (upper blue banner) of the integrative model include location (*latitude* and *longitude*), time (Doy, day of year; Hour, simulated time of the day; DAA, days after anthesis), climate data (*R_s_*, total solar radiation; *VPD*, difference in water vapor pressure of air; *T_a_*, air temperature; *RH*, relative humidity of air; *CO_2_*, atmospheric CO_2_ concentration) and agronomic practice (*θ*, soil water content of the root zone; *N*, soil nitrogen content of the root zone). The tomato canopy is assumed to be a ‘big leaf’ whose exchange of gaseous water and CO_2_ are determined by stomatal conductance (*g_sNCO2_*), which thus integrates the environmental data. Information on *g_sNCO2_* feeds into the plant water module (blue dashed outline), and the plant carbon module (green dashed outline). It is assumed that the amount of carbon loaded to phloem from the leaf equals the amount of carbon unloaded to the stem, root, and fruit from phloem, and the carbon unloading rates for these organs depend on the sucrose concentration in phloem sap (*Cp*) and their respective sink activity. In the fruit module (orange dashed outline), water enters the fruit from phloem/xylem, driven by the water potential gradient between the stem and the fruit. Thus, the fruit module is coupled with the plant water and carbon modules by water potential gradient and *Cp*. Model outputs are fresh and dry weight of organs and the concentrations of soluble sugar and starch in fruit.

**Figure 2:**
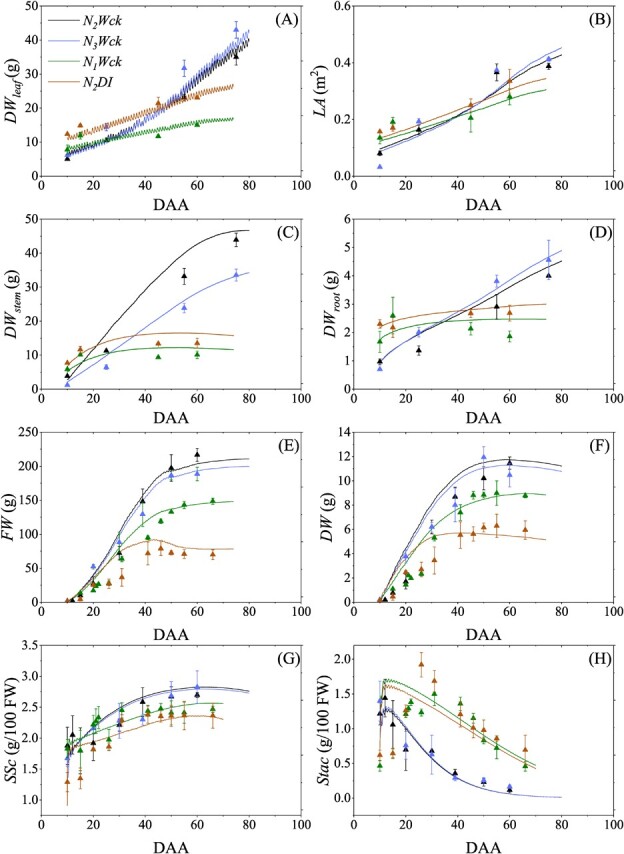
Calibration and validation of the integrative TGFS model with different N and irrigation inputs. Observed (triangles) and simulated (curves) dynamics of (**A**) leaf dry weight (*DW_leaf_*), (**B**) leaf area (*LA*), (**C**) stem dry weight (*DW_stem_*), (**D**) root dry weight (*DW_root_*), (**E**) individual fruit fresh weight (*FW*), (**F**) individual fruit dry weight (*DW*), (**G**) fruit soluble sugar concentration relative to FW (*SSc*), and (**H**) starch concentration relative to FW (*Stac*) in days after anthesis (DAA) throughout the plant growing season. Symbols and error bars represent means and standard deviations of the measurements (n = 3–5), respectively. The model was calibrated against measurements from plants treated with moderate N application and full irrigation (N2Wck). The model was validated against measurements from plants treated with high amounts of N and full irrigation (N3Wck), low amounts of N and full irrigation (N1Wck), or moderate amounts of N with an irrigation deficit (N2DI).

The integrative TGFS model (Fig. 1) was calibrated against measurements of real tomato plants grown under optimal nitrogen and water conditions (N2Wck in Experiment A). Calibration results show that the observed plant growth variables, including *DW_leaf_*, *LA*, *DW_stem_*, and fruit development variables, such as *FW*, *DW*, and fruit soluble sugar concentration (*SSc*), were well simulated as the RRMSE values range from 5.57% to 18.59% (Fig. 2A–G; Table 1). The relatively high RRMSE for fruit starch concentration can mainly be attributed to the scatter in the observations and the very low values late in fruit development. However, the dynamic trend during fruit development estimated by the model has a small MAE which is deemed satisfactory (Fig. 2H; Table 1). Additionally, the simulated *C_str_*, *C_ns_*, *SS*, *Sta*, *g_sNCO2_*, *ψ_stem_*, *Tr*, *Pn*, *Cp* and respiration of the leaf, stem, root, and fruit (Fig. S1, see online supplementary material) are consistent with previously reported values [26, 28, 39, 55, 56].

**Table 1:** Goodness of fit estimated by mean absolute error (MAE) and relative root mean square error (RRMSE) between the measured and simulated tomato leaf dry weight (*DW_leaf_*), leaf area (*LA*), stem dry weight (*DW_stem_*), root dry weight (*DW_root_*), individual fruit fresh weight (*FW*), and dry weight (*DW*), as well as concentrations of soluble sugar and starch in fruit (*SSc* and *Stac* respectively) for pot-grown tomato treated with different amounts of N and water. The units MAE in the table are the same as those of the corresponding variables.

Goodness of fit	Variable	N2Wck	N3Wck	N1Wck	N2DI
MAE	*DW_leaf_* (g)	1.01	2.48	3.38	3.34
	*LA* (m^2^)	0.06	0.04	0.07	0.08
	*DW_stem_* (g)	10.87	4.51	8.06	6.10
	*DW_root_* (g)	0.51	0.58	0.84	0.86
	*FW* (g)	14.00	16.24	15.41	18.32
	*DW* (g)	1.88	2.05	2.20	2.75
	*SSc* (g/100 g FW)	0.23	0.29	0.24	0.26
	*Stac* (g/100 g FW)	0.20	0.23	0.48	0.52
RRMSE	*DW_leaf_* (g)	5.6%	10.2%	11.6%	10.8%
	*LA* (m^2^)	8.5%	10.1%	13.1%	20.8%
	*DW_stem_* (g)	15.7%	18.3%	26.1%	20.9%
	*DW_root_* (g)	7.8%	10.9%	15.1%	12.9%
	*FW* (g)	10.3%	13.3%	21.8%	28.3%
	*DW* (g)	13.8%	15.4%	21.4%	25.7%
	*SSc* (g/100 g FW)	18.6%	15.8%	14.3%	14.8%
	*Stac* (g/100 g FW)	21.1%	28.9%	35.5%	44.8%

Note: Datasets from N2Wck in Experiment A were used for calibration. Datasets from N3Wck in Experiment A, N1Wck and N2DI in Experiment C were used for validation.

To validate the model, it was tested against three independent datasets of measurements from plants treated with different amounts of N and water, namely N3Wck in Experiment A and N1Wck and N2DI in Experiment C (see Materials and Methods for details of treatments). For these three growth conditions, the dynamics of *DW_leaf_*, *LA*, *DW_stem_*, *DW_fruit_*, *FW*, *DW*, and *SSc* were simulated reasonably well (RRMSE 10.1% to 28.3%, Table 1) with the TGFS model (Fig. 2A–G). The fruit starch concentration simulation for N3Wck conditions is acceptable, as the MAE is 0.23 g 100 g^−1^ FW and the RRMSE is 28.9%, similar to the values for the N2Wck condition. However, the predictions of *FW*, *DW*, *SSc*, and *Stac* during the first phase of fruit development were not completely satisfactory for stressed tomatoes (N1Wck and N2DI in Fig. 2E–H), which might be because the predicted *ψ_stem_* can’t fully reflect the deficit status of the plant-fruit system and the early deviation of the measured *Stac* peak. Overall, the integrative model efficiently simulated the development of the leaf, stem, root, and fruit, and the dynamics of soluble sugar and starch in the fruit, by comprehensively considering the effects of environmental factors. Thus, TGFS could be used to predict plant behaviour and fruit sugar responses under different environments.

### Future climate change will improve fruit size but not sugar concentration

**Figure 3:**
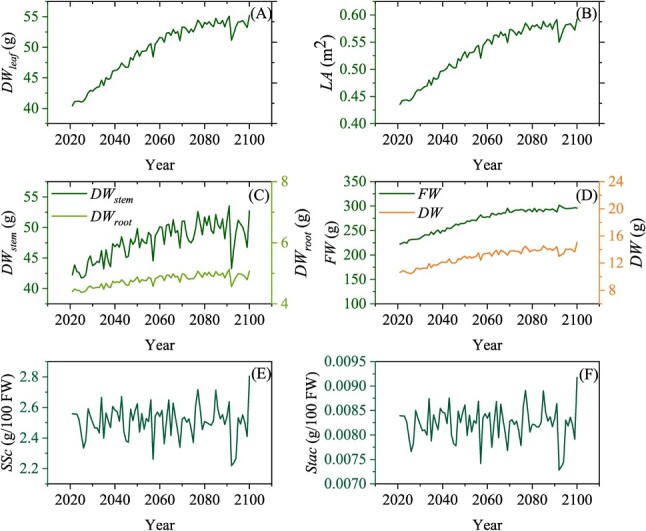
Simulations of tomato (**A**) leaf dry weight (*DW_leaf_*), (**B**) leaf area (*LA*), (**C**) dry weight of stem (*DW_stem_*) and root (*DW_root_*), (**D**) individual fruit fresh (*FW*) and dry weight (*DW*), (**E**) soluble sugar concentration (*SSc*), and (**F**) starch concentration (*Stac*) of mature fruit from 2021–2100 under the simulated changing climate. For each trait, the average value in the last 5 days of each growing season is taken as the final value of that season.

We applied the TGFS model to assess how tomato growth and fruit quality would respond to increasing air temperature (*T_a_*) and increasing atmospheric CO_2_ concentration (*CO_2_*), characteristic of a climate change scenario for the location where the experimental data were collected, and assuming the current moderate N application and full irrigation remained the same over the predicted period (2021–2100) (Fig. 3).

With continuous rises in *T_a_* and *CO_2_*, the dry weights of the modelled leaf, stem and root show significant logistic upward trends (Fig. 3). Compared to the start of the simulated period (current conditions), the simulated dry weights of the leaf, stem and root increased by 36.5%, 24.9%, and 15.2% (Fig. 3A and C), respectively, by the end of the simulation period. The simulated carbon composition in the leaf revealed that both *C_str_* and *C_ns_* (Fig. S2, see online supplementary material) showed similar trends over the period, increasing by 36.0% and 44.6%, respectively. Accordingly, the predicted plant *LA* increased from 0.44 m^−2^ to 0.59 m^−2^, a 36.0% increase (Fig. 3B). The larger increases in *C_ns_* compared to *C_str_* in the leaf suggests that the tomato leaf would accumulate proportionally more non-structural carbon, potentially providing more materials and energy to support tomato growth under future climates. The enhancement of plant carbon assimilation due to enlarged leaf and higher photosynthesis rates (Fig. S3A, see online supplementary material) would improve the growth of other organs. Consistent with this, over the simulation period significant logistic increases were observed from 222.24 g to 295.95 g for the *FW* and from 10.62 g to 15.06 g for the *DW* of individual fruit (Fig. 3D). Meanwhile, the average stomatal conductance over each year (*g_sNCO2__m*) decreases from 0.17 to 0.13 mol H_2_O m^−2^ s^−1^ and water consumption by leaf transpiration (*Tc*) decreases from 84.64 to 82.98 L/plant/season with the increase in *CO_2_* and air temperature (Fig. S3 D, E, F, see online supplementary material).

Although beneficial climate impacts on tomato plant and fruit growth may be seen in the future, the taste quality of the ripe fruit may not improve (Fig. 3E and F). From 2021 to 2100, the concentrations of soluble sugar in mature fruit (*SSc*) showed a slight downward trend at the rate of 3.7 × 10^−5^ g/100 g FW/year. No obvious trends were found in the simulated starch concentration in mature fruits (*Stac*). Fruit *SSc* is related to carbon and water fluxes into and out of the fruit. For fruit, the annual net influx of carbon (*FruitC_net_*) was slightly lower than that of water (*FruitW_net_*) (Fig. S3G, see online supplementary material), both are positively related to CO_2_ and air temperature (Fig. S3 H and I, see online supplementary material). Additionally, the estimated inter-annual variability in soluble sugar in mature fruit in the future is large, with a variance of 1.2%, suggesting that fruit quality variation between year may become higher.

Overall, simulation results show that future increases in *T_a_* and *CO_2_* concentration would promote growth of tomato leaf, stems, roots, and fruit. However, there is little evidence that the bigger fruit would be better quality with more concentrated soluble sugar.

### Reducing nitrogen and water application could obtain bigger and sweeter tomato fruit in the future

Considering the objective to reach carbon neutrality and dealing with the current context of water scarcity and excessive nitrogen fertilisation [7, 31], sustainability scenarios were designed to reduce inputs of nitrogen and water.

To explore whether modifying N application or irrigation would produce better tomato fruit, the responses of mature tomato fruit FW and soluble sugar concentration to different N and water management practices were investigated through the validated TGFS model with inputs reflecting future climate change. Final N and irrigation scenarios were compared to initial reference N2Wck conditions, as shown in Eqn. 1

(1) \begin{equation*} \varDelta =\frac{NiW{i}_{\left[ final\right]}-N2 Wc{k}_{\left[ initial\right]}}{N2 Wc{k}_{\left[ initial\right]}}\times 100 \end{equation*}

where $\varDelta$ refers to changes in the *FW* or *SSc* of mature tomato fruit in different N and water scenarios and *i* refers to a specific N treatment and irrigation (W). $NiW{i}_{\left[ final\right]}$ is the average value of *FW* or *SSc* of mature fruit in the last five years of 2021–2100 (e.g. 2096–2100, to avoid inter-annual uncertainty) under $NiWi$ scenarios. $N2 Wc{k}_{\left[ initial\right]}$ is the value of *FW* or *SSc* of mature fruit in 2021 in the N2Wck condition.

**Figure 4:**
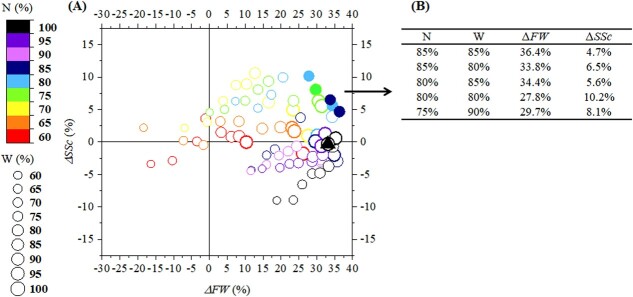
Changes in the mature tomato fruit fresh weight (*∆FW*, %) and soluble sugar concentration (*∆SSc*, %) between 2021 and 2100 according to various possible sustainable scenarios using less nitrogen (N) and water (W) under future climate change. **A** The position of bubbles in the figure shows the percentage difference in *FW* and *SSc* at the end of the different scenarios and their respective values in the reference condition in 2021 (N2Wck), which can be expressed as$\varDelta =\frac{NiW{i}_{\left[ final\right]}-N2 Wc{k}_{\left[ initial\right]}}{N2 Wc{k}_{\left[ initial\right]}}\times 100$, where $\varDelta$ refers to percentage differences in *FW* or *SSc* of mature tomato fruit in different N and water scenarios, $NiW{i}_{\left[ final\right]}$ is the average of the five values of *FW* or *SSc* of mature fruit from 2096 to 2100 under $NiWi$, and $N2 Wc{k}_{\left[ initial\right]}$ is the value of *FW* or *SSc* of mature fruit in 2021 for the N2Wck condition. The average value of the last five years of the simulation was used as the endpoint to avoid interannual uncertainty. N and water input levels relative to the reference level are represented by the bubble size and color as shown in the legend. Change in *FW* and *SSc* in the future under the reference condition is marked with a filled black triangle. **B** Values for selected sustainable scenarios highlighted in (**A**) by filled circles.

Fig. 4A shows the percentage difference in *FW* and *SSc* caused by different N and water treatments from 2021 to 2100. When N application is relatively high (90% to 100%), a decrease in irrigation reduces the benefit of the future climate change on the *FW*, limiting the *FW* percentage increase to between 11.7% and 35.7% in the future, with no significant increase in sugar content. When N application was at a moderate level (75% to 85%), a mild water deficit increases *SSc* by 10.6% while maintaining a positive effect on the *FW*. However, severe water deficit cancels out the positive effect of climate change on the *FW*. When N application is low (60% to 70%), *FW* and *SSc* fall to an unfavourable level, with water deficit becoming stressful. The 40% reduction in N and water application could reduce tomato *FW* by 16.3% and *SSc* by 3.4% by 2100.

To find N and water combinations optimal for obtaining bigger and sweeter fruits in future climate conditions, the Pareto front was considered. Five combinations were selected on this front (Fig. 4A and B). The results indicate that by the end of this century, decreasing N application from 15% to 25% while decreasing water supply from 10% to 20% could increase the FW of mature tomato fruit from 27.8% to 36.4% and the soluble sugar content from 4.7% to 10.2%.

## Discussion

The integrative TGFS model developed here provides a detailed dynamic representation of the growth of the tomato leaf, stem, root, and fruit in the post-flowering stage, in addition to computing the soluble sugar and starch concentrations in fruit. The TGFS model was coupled to the N-CO_2_-Jarvis model to estimate tomato stomatal conductance (*g_sNCO2_*), which is a key factor controlling leaf gas exchange and hence water and carbon balance [36, 37]. As the N-CO_2_-Jarvis model considers various environmental variables (radiation, temperature, VPD, soil water content, soil N content, and atmospheric CO_2_ concentration) on leaf gas exchange, the resulting integrative model is enhanced. As demonstrated here, the model can be used to investigate the interactions of the environmental conditions with agronomic practices on tomato growth and fruit quality. The TGFS model simulates vegetative and reproductive growth and links the growth of plants and fruits through water and carbon fluxes controlled by *ψ_stem_* and *Cp*. According to the validation against measurements from real tomato plants, the integrative model accurately simulates tomato growth and fruit sugar concentrations.

Simulation results from the integrative model suggest that climate change over the next century would produce an overall increase in tomato growth if current water and N management was continued, which is consistent with some previous findings [5, 57–59]. However, under this scenario, no positive effects on fruit sugar concentrations are predicted. When *CO_2_* increases from 413.38 μmol mol^−1^ to 538.36 μmol mol^−1^ and *T_a_* increases from 7.41°C to 8.83°C, the simulated *FW* increases by 33.2%, which is consistent with a range of 19.65% to 43% increment of *FW* founded in similar combined *CO_2_* × *T_a_* controlled experiments [60, 61]. Increases in *CO_2_* and *T_a_* would increase the leaf photosynthetic rate significantly (Fig. S3B and C, see online supplementary material), providing more energy and materials for tomato growth [1–3]. However, future downward trends in stomatal conductance and plant transpiration accumulation (Fig. S3D, see online supplementary material), caused by the increasing *CO_2_* and *T_a_* (Fig. S3E and F, see online supplementary material), indicate that stomatal and non-stomatal controls would co-ordinately maximise leaf photosynthesis [62] and save more water from transpiration. *FruitW_net_* values higher than *FruitC_net_* values (Fig. S3G, see online supplementary material) indicate that *SSc* would tend to decrease slightly in the future (Fig. 3E), which may be due to the fruit accumulating more water. No significant difference or a slight decrease of tomato fruit soluble sugar concentration were also found in the tomato fruit grown under controlled condition of *CO_2_* × *T_a_* [60, 61].

It should be noted that other simulation results show a decrease in crop growth under future climate change ([63]). These obvious growth reductions are partly attributed to the extreme RCP scenarios studied and to worse agronomic conditions, such as less irrigation. In [5] the impact of climate change on grape growth and quality and planting area was reviewed, highlighting the complexity of future predictions, which may be specific to the local environmental conditions, the type of crop and the crop model used [6]. Although current simulation studies focus mainly on future crop yield, evidence of the negative effects of future climate change on fruit quality can be found in a few studies [63, 64]. Agronomic strategies will become a key factor for the future sustainability of crop production [8, 63]. With our integrative model, several candidate strategies for soil water and nitrogen levels were identified that represent a good compromise between tomato growth and fruit quality. Reducing N and water supply would affect mature *FW* and *SSc* in the future. When the amount of N applied is at a relatively high level, inadequate water input induces an imbalance between plant N and water relations [65], limiting physiological processes such as transpiration and growth [66]. As we found (Fig. 4A), the consequence is that part of the percentage increase in *FW* is offset with lowers *SSc* under future climates. When the N is reduced to moderate levels, a mild decrease in water supply likely promotes sugar transport mechanisms and sugar accumulation in berries when carbon assimilation by leaves is slower [14, 43]. As the intensity of water deficit gradually increases, the corresponding increase in *SSc* (Fig. 4A) could be due to dehydration because less water accumulates in the fruit [25]. Nevertheless, some positive trends in *FW* and *SSc* are found in some of our scenarios. According to our model, severe N and water deficits cannot support a continued increase in tomato *FW* and *SSc* in the future, as plant growth and sugar accumulation in fruits would be limited under such conditions [65]. It should be noted that the response of stomata and growing leaves to soil N content decreasing from supra-optimal to sufficient to deficient could be biphasic, as a previous study [67] and the coupled N-CO_2_-Jarvis model shows [35]. Therefore, in some cases, increases in *FW* under particular N and water management scenarios in the future could be slightly higher than those observed under the probably sub-optimal N2Wck condition. In the context of future climate change, limiting the increase in the *FW* of mature fruit might significantly improve *SSc*. For example, by reducing N and water supply by 20% in the future, the *SSc* of mature tomato fruits may increase by 10.2% compared to *SSc* values for fruit grown in 2021 conditions and a 27.8% increase in *FW* can be expected by 2100. Previous studies indicate that the amount of irrigation needed for tomato in the future can be reduced by 5%–40% without a significant loss in yield [9, 63]. Our results show that this trade-off between tomato FW and soluble sugar concentration could be regulated by N and water input management in the future.

Overall, the TGFS model proved its ability to simulate plant growth and the fruit sugar metabolism of tomato grown in a changing environment, associated with the climate change or with varying agronomical practices. However, the model was greatly simplified as the tomato canopy and fruit were merely assumed to be a ‘big leaf’ and a ‘big truss’, respectively. Connecting our TGFS model with a dynamic structural model may be helpful for characterising the development of the tomato canopy structure under different conditions and any subsequent effects on carbon assimilation, growth, and fruit sugar metabolism. This would give a more nuanced picture of tomato growth. In addition, the current version of the TGFS model has some shortcomings that suggest useful ideas for model improvement, mainly including the following: (i) the Jarvis-type of stomatal conductance models didn’t consider the interactive effects of external factors on stomatal conductance, which could lead to inaccuracy in simulating the photosynthesis and transpiration; (ii) water and nitrogen stress could affect sink activity and the priority of plant carbon allocation; for example, sever water/nitrogen stress may affect flowering, fruit set, and lead to a preferential carbon allocation to the root, and the current TGFS model does not account for these responses; and (iii) further studies of photosynthetic biochemical reactions can enrich plant carbon flux modelling, as the non-structural carbon accumulated in the leaf may have a feedback effect on photosynthesis.

##  

In summary, the whole tomato plant model TGFS we developed in this study considers the impact of nitrogen and CO_2_ on interactions between the plant, the fruit, management and the environment, performed well by closely simulating the tomato leaf, stem, root, and fruit growth, as well as fruit soluble sugar and starch concentrations with different irrigation and nitrogen input values. Climate change is predicted to generally have positive effects on tomato growth but not on soluble sugar concentration in the fruit. However, reducing nitrogen input by 15%–25% and irrigation input by 10%–20% from today’s baseline may produce bigger and sweeter tomatoes in the future.

## Materials and methods

### Model integration

We developed an integrative model of tomato plant and fruit growth and fruit sugar metabolism, named TGFS (Fig. 1). The tomato model is based on an explicit description of plant-fruit fluxes of water and carbon that takes into account biological responses to environmental factors and agronomic practices during the post-flowering stages of tomato. In this model, the whole-plant is conceptually divided into four compartments: leaf, stem, root, and fruit. The tomato canopy was assumed to act like a ‘big leaf’, carrying out transpiration and photosynthesis. Fruits were assumed to grow on a ‘big truss’, growing homogeneously.

The TGFS model integrates a plant water module, a plant carbon module and a fruit module (Fig. 1). For the water resource, water fluxes are driven by gradients in water potential. Water enters the plant from the soil by root absorption and dissipates into the atmosphere mainly through leaf transpiration. The flux of water to the fruit is driven by the difference in water potential between the stem and the fruit. In the plant carbon module, the Farquhar model is used to calculate the carbon assimilation by the canopy [33]. The improved N-CO_2_-Jarvis stomatal conductance model [34, 35] was on the one hand used to control the leaf transpiration, and on the other hand it was coupled to the Farquhar model to reflect the effects of weather factors, soil water and N content, and atmospheric CO_2_ concentrations on leaf carbohydrate production. Carbohydrates synthesised in the leaf are loaded into the phloem as sucrose, then allocated to the stem, root and fruit. In the fruit module, the water and carbon fluxes to the fruit are related to the stem water potential (*ψ_stem_*) and concentration of sucrose in the phloem solution (*Cp*), thus linking plant and fruit growth. Tomato fruit growth was simulated with the fruit growth model [22] and tomato sugar metabolism was modelled with the fruit sugar model TOM-SUGAR [25]. The details of each module are presented in more detail in the following sections.

### The N-CO_2_-Jarvis stomatal conductance model

The canopy, essentially a “’big leaf’, is the main site of CO_2_ and water vapour exchange, which are both governed by stomatal conductance [36, 37]. Therefore, stomatal conductance was considered as a breakthrough point to reflect the effects of meteorological factors, soil water, soil N, and CO_2_ concentration on gas exchange. Here, stomatal conductance was simulated using the N-CO_2_-Jarvis model adapted from [34, 35].

(2) \begin{equation*} {g}_{sNCO2}={g}_{s\max}\times f\left({R^{\hbox{'}}}_n\right)f\left({T}_a\right)f(VPD)f\left(\theta \right)f(N)f\left(C{O}_2\right) \end{equation*}

In this expression, *g_smax_* is the maximum leaf stomatal conductance (mol H_2_O m^−2^ s^−1^); *g_sNCO2_* is the actual stomatal conductance after considering the effects of net solar radiation intercepted by the canopy (*R_n_′*, MJ m^−2^ h^−1^), air temperature (*T_a_*,°C), VPD (*VPD*, kPa), soil water content (*θ*, cm^3^ cm^−3^), soil mineral N content (*N*, mg kg^−1^), and atmospheric CO_2_ concentration (*CO_2_*, μmol mol^−1^). The reduction functions of the N-CO_2_-Jarvis stomatal conductance model are presented in Method S1 (see online supplementary material). Model variables are summarised in Table S1 (see online supplementary material) and the details of model parameters are shown in Table S2 (see online supplementary material).

### Plant water module

The leaf transpiration rate (*Tr*, g h^−1^) was calculated based on *g_sNCO2_* and related to *VPD* and leaf area (*LA*) [38] as follows:

(3) \begin{equation*} Tr= VPD\times {g}_{sNCO2}\times LA \end{equation*}

*Tr* is assumed to be equal to the rate of water absorption by the roots, driven by the water potential gradient between the soil and stem. Soil water potential (*ψ_soil_*, MPa) was estimated from the soil water content (hourly dynamics given as model input) according to a characteristic soil moisture curve. Thus, the stem water potential (*ψ_stem_*, MPa) was obtained as follows,

(4) \begin{equation*} {\psi}_{stem}={\psi}_{soil}- Rp\times Tr \end{equation*}

where *Rp* was the hydraulic resistance from the soil to the tomato stem (MPa h g^−1^). *Rp* was estimated from sap-flow measurements *ψ_stem_* and *ψ_soil_* by [39] as presented in Fig. S4 (see online supplementary material).

### Plant carbon module

To simulate the leaf photosynthesis (*Pn*), *g_sNCO2_* was coupled with the Farquhar model [33, 40]. Negative linear relationships between plant age and the Farquhar parameters *Vcmax* and *Jmax* were detected over the simulation period and included in the modelling (Fig. S5, see online supplementary material). The carbon in the leaf compartment (*C_t_*, g C) is composed of the structural carbon (*C_str_*, g C) and non-structural carbon (*C_ns_*, g C).

The change in the *C_str_* pool is dependent on its size and on the availability of non-structural carbon [41], so the size of this carbon sink was calculated according to:

(5) \begin{equation*} \frac{d{C}_{str}}{dt}={C}_{str}\times {K}_{ml}\times \frac{C_{ns}}{C_{ns}+{C}_{str}} \end{equation*}

where *K_ml_* is the maximal relative accumulation of structural carbon mass in the leaf (h^−1^).

The amount of the non-structural carbon pool in the leaf (*C_ns_*) is determined by the overall carbon balance between leaf photosynthesis, carbon loaded into the phloem, consumption due to respiration, and structural growth. Thus, the change in *C_ns_* was described as:

(6) \begin{equation*} \frac{d{C}_{ns}}{dt}= Pn\times LA- Loadin{g}_{leaf}- Mres{p}_{leaf}-\frac{d{C}_{str}}{dt} \end{equation*}

where *Loadingleaf* is the loading rate of carbon into phloem and *Mrespleaf* is the leaf respiration rate (g C h − 1). *LA* is calculated from *Cstr* and the specific leaf area of structural carbon (*SLAs*, Table S2, see online supplementary material).

(7) \begin{equation*} LA={C}_{str}\times SLAs \end{equation*}

The carbon consumed by leaf respiration (*Mrespleaf*, g C h − 1) includes maintenance respiration and growth respiration,

(8) \begin{align*} Mres{p}_{leaf}={q}_{mleaf}D{W}_{leaf}{Q_{10}}^{\left({T}_a-20\right)/10}+{q}_{gleaf}\frac{d{C}_{str}}{dt} \end{align*}

where *DW_leaf_* is the leaf dry weight (g), and *Q_10_* is a temperature sensitivity coefficient for maintenance respiration, while *q_mleaf_* and *q_gleaf_* are the coefficients for leaf maintenance and growth respiration, respectively (Table S2, see online supplementary material).

The carbon loading rate into the phloem is related to the carbon loading capacity of the leaf itself and of the leaf non-structural carbon content [42, 43]. Here we used the following equation to describe the carbon loading rate:

(9) \begin{equation*} Loadin{g}_{leaf}= LA\times {V}_{\mathrm{maxleaf}}\times \frac{C_{ns}/\left({C}_{ns}+{C}_{str}\right)}{K_{m leaf}+{C}_{ns}/\left({C}_{ns}+{C}_{str}\right)} \end{equation*}

where *V_maxleaf_* is the maximum leaf carbon loading rate per unit *LA* and *K_mleaf_* was the Michaelis–Menten constant for leaf carbon loading [28] (Table S2, see online supplementary material).

The leaf dry weight can be calculated as:

(10) \begin{equation*} D{W}_{leaf}=\frac{C_{ns}+{C}_{str}}{c_{leaf}} \end{equation*}

where *c_leaf_* is the carbon amount in 1 g of leaf dry mass, which has the value of 0.36 g C (g DW)^−1^ for tomato leaves [44].

Carbon loaded from the leaf to the phloem is then allocated to the stem, root, or fruit. Carbon unloaded from the phloem by the stem and root is used for growth and respiration. The rates of carbon unloaded by the stem and root (*Uptake_stem_* and *Uptake_root_*) are related to the sink size (carbon content of stem *C_stem_* and carbon content of root *C_root_*, both in g C), sucrose concentration in the phloem solution (*Cp*), and plant developmental stage. Carbon uptake by the stem and root are expressed as:

(11) \begin{align*} Uptak{e}_x={C}_x\times \frac{K_{p\to x}}{1+\exp \left({A}_{p\to x}\times \left(t-{B}_{p\to x}\right)\right)}\times Cp \end{align*}

where the subscript *x* indicates variables related to either the stem or the root; *K_p_* is the maximum rate coefficient of stem or root growth; *A_p_* is the attenuation coefficient of the stem or root growth rate; and *B_p_* is the time coefficient of stem or root growth rate attenuation, detailed in Table S2 (see online supplementary material).

Changes in carbon amounts in the stem or root during plant growth can be expressed as follows:

(12) \begin{equation*} \frac{d{C}_{\mathrm{x}}}{dt}= Uptak{e}_x- Mres{p}_x \end{equation*}

(13) \begin{equation*} Mres{p}_{\mathrm{x}}={q}_{mx}D{W}_x{Q_{10}}^{\left({T}_a-20\right)/10}+{q}_{gx}\frac{d{C}_x}{dt} \end{equation*}

The dry weight of the stem or root (*DW_x_*) can be calculated from the total amount of carbon in the organ and the amount of carbon in 1 g of the respective dry mass (*c_x_*, g C (g DW)^−1^; Table S2, see online supplementary material).

(14) \begin{equation*} D{W}_{\mathrm{x}}=\frac{C_x}{c_x} \end{equation*}

### Fruit module

The tomato fruit is mainly a carbon sink. Carbon allocated to fruit is used for fruit growth and respiration while soluble sugars and starches accumulate (Fig. 1). Thus, the tomato fruit growth model [22] and the fruit sugar model (TOM-SUGAR) [25] were coupled in the integrative TGFS model to simulate the dry weight (*DW*) and fresh weight (*FW*) of individual fruit and the concentrations of soluble sugar and starch they contain. The plant water and carbon modules simulate the fruit module inputs, which are stem water potential (*ψ_stem_*) and sucrose concentration in the phloem solution (*Cp*). The carbon absorbed by the fruit can be described as follows:

(15) \begin{equation*} Uptak{e}_{fruit}={U}_s\times {c}_{suc}\times Fn \end{equation*}

where *U_s_* is the rate of sucrose input from the phloem to the fruit (g h^−1^), whether by active transport, mass flow or passive diffusion (detailed in Method S2 (see online supplementary material) and [17, 22]). *Fn* is the number of fruits on one plant. Details of how the fruit growth model is coupled to the TOM-SUGAR model are presented in Methods S2 (see online supplementary material). The parameters used in the fruit module are summarised in Table S3 (see online supplementary material).

The amount of carbon loaded from the leaf was assumed to be equal to the amount of carbon unloaded by the stem, root, and fruit.

(16) \begin{equation*} Loadin{g}_{leaf}= Uptak{e}_{stem}+ Uptak{e}_{root}+ Uptak{e}_{fruit} \end{equation*}

The sucrose concentration in phloem sap, *Cp*, can be obtained at the whole plant level by solving the combination of equations 5–16.

### Experimental conditions

Experimental data were used to calibrate and validate the models. The data were collected from experiments where indeterminate tomato plants (*Lycopersicon esculentum*, cv. Jinpeng 11) were grown in pots seasonally provided with different amounts of water and N in the summer of 2015 (Experiment A), the summer of 2016 (Experiment B) and the winter of 2016–2017 (Experiment C). Experiments included low, moderate, and high N application under full irrigation (N1Wck, N2Wck, and N3Wck) and moderate N application with whole-season irrigation deficit (N2DI). Five trusses of pot-grown tomato were kept after transplantation. Plant age was expressed as days after anthesis (DAA). During the experiment, meteorological factors, soil water content, soil mineral N content, and plant variables such as leaf area, organ dry mass, leaf gas exchange, and plant transpiration were measured. Experimental design and measurements are detailed in Method S3 (see online supplementary material). Flowers on the second and third trusses were marked with their pollination date. Samples or measurements were taken from fruits marked with similar pollination dates, assuming these fruits were of the same age and constituted the ‘big truss’ compartment. Quality traits such as fresh and dry weight of fruit and their soluble sugar and starch contents were measured from 10 days after anthesis at intervals of 5–10 days.

### Model input and initial conditions

The integrative model is driven by meteorological factors, including solar radiation (*R_s_*), air temperature (*T_a_*), relative humidity (*RH*), atmospheric CO_2_ concentration (*CO_2_*), and factors related to agronomic practices including soil water content (*θ*) and soil mineral nitrogen content (*N*). To initialise the model, the dry weight of the leaf, stem, and root, the structural and non-structural carbon fractions in the leaf, individual fruit FW, DW, and total soluble sugar and starch concentrations in fruit are required as starting values for the simulation.

### Model calibration and validation

The Farquhar and N-CO_2_-Jarvis models were calibrated using a dataset of meteorological factors, soil water, soil N content, leaf area, and leaf stomatal conductance obtained in Experiment C. The N-CO_2_-Jarvis model parameters were estimated with the *optim* function in R using the *Nelder–Mead* method. Farquhar model parameters *Jmax* and *Vcmax* were fitted by the *fitacis* function in the ‘*plantecophys*’ R package with measured A-Ci curves (photosynthesis rate at varying CO_2_ concentrations) obtained from Experiment B using the *bilinear* method. The photosynthesis rate was calculated using the *Photosyn* function in the ‘*plantecophys*’ R package.

The parameters of the plant and fruit carbon modules were calibrated at the whole-plant scale, including parameters of plant growth (*K_ps_*, *A_ps_*, *K_pr_*, *A_pr_*), fruit growth (*v_m_*, t*, *τ*, *axp*, *k_phi_*, *τ_s_*), and fruit sugar metabolism (*λ*, *n*, *k_5_*, *k_5m0_*, *u_5m_*, *τ_5m_*) using the data related to meteorological factors, soil water, soil N content, organ dry weight, and fruit sugar concentrations from N2Wck in Experiment A.

Datasets from N3Wck in Experiment A and N1Wck and N2DI in Experiment C were reserved for validation of the integrative TGFS model. A genetic algorithm was applied to search for the best parameter combination that minimised the objective criterion in Eqn. 17 [45] implemented with the *ga* function in the R package ‘*GA*’ [46].

(17) \begin{equation*} {\displaystyle \begin{array}{l} criterion=\frac{\sum {\left({y}_{oDWl}-{y}_{sDWl}\right)}^2}{{\operatorname{var}}_{oDWl}}+\frac{\sum {\left({y}_{oDWs}-{y}_{sDWs}\right)}^2}{{\operatorname{var}}_{oDWs}}+\frac{\sum {\left({y}_{oDWr}-{y}_{sDWr}\right)}^2}{{\operatorname{var}}_{oDWr}}+\\ {}\frac{\sum {\left({y}_{oDWf}-{y}_{sDWf}\right)}^2}{{\operatorname{var}}_{oDWf}}+\frac{\sum {\left({y}_{oFWf}-{y}_{sFWf}\right)}^2}{{\operatorname{var}}_{oFWf}}+\frac{\sum {\left({y}_{osol}-{y}_{ssol}\right)}^2}{{\operatorname{var}}_{osol}}+\frac{\sum {\left({y}_{osta}-{y}_{ssta}\right)}^2}{{\operatorname{var}}_{osta}}\end{array}} \end{equation*}

*y_oDWl_*, *y_oDWs_*, *y_oDWr_*, *y_oDWf_*, *y_oFWf_*, *y_osol_*, and *y_osta_* are the experimentally observed values for leaf dry weight, stem dry weight, root dry weight, fruit dry weight, fruit fresh weight, fruit soluble sugar content, and fruit starch content, respectively. *y_sDWl_*, *y_sDWs_*, *y_sDWr_*, *y_sDWf_*, *y_sFWf_*, *y_ssol_*, and *y_ssta_* are their respective simulation values. *var_oDWl_*, *var_oDWs_*, *var_oDWr_*, *var_oDWf_*, *var_oFWf_*, *var_osol_*, and *var_osta_* are the variances of the observed values.

To solve the integrative model, the *ode* solver in the R package ‘*deSolve*’ [47] was applied for the numerical computation. The mean absolute error (MAE) and relative root mean squared errors (RRMSE) of plant growth and fruit sugar concentrations were calculated to evaluate the goodness of fit of the model using Eqn. 18 and 19 respectively [48]. All data analyses, parameter estimation, and model solving were performed using R software [49].

(18) \begin{equation*} MAE=\frac{1}{m}\sum \limits_{j=1}^m\left|{y}_{oj}-{y}_{sj}\right| \end{equation*}

(19) \begin{equation*} RRMSE=\frac{\sqrt{\frac{1}{m}\sum \limits_{j=1}^m{\left({y}_{oj}-{y}_{sj}\right)}^2}}{\frac{1}{m}\sum \limits_{j=1}^m{y}_{oj}} \end{equation*}

### Future climate conditions and scenario simulations

To predict how plant growth and fruit quality might respond to future climate changes, we implemented the integrative TGFS model to take future climate change into account. Possible future climate situations were obtained using a statistical downscaling method on a CNRM-CM5 climate model [50, 51]. Simulated future data is downscaled based on the observed data of the Shiyang River Basin meteorological station where our experiments were conducted. The predicted future climate data is in an RCP4.5 medium emission scenario [52], the most likely and representative future scenario [53, 54], with CO_2_ concentration at 550 ppm by 2100. Temperature data was interpolated from a day scale to an hour scale based on sinusoidal function and the occurrence time of the maximum and minimum temperature is set as 1 p.m. and 1 a.m. The future air temperatures and CO_2_ concentrations are shown in Fig. S6 (see online supplementary material).

Simulations were run with the integrative model using settings to reflect future climate and to explore the possible effects of irrigation and nitrogen application on tomato growth and fruit sugars in those conditions. Given previous results [39], N2Wck was set as the reference condition for N and water application. Thus, in the sustainability scenarios, the amounts of N and water input were decreased from 100% to 60% of N2Wck levels at 5% intervals, thus giving nine levels for each variable and a total of 81 scenarios. The N and water input in these scenarios are N2Wck values multiplied by coefficients corresponding to the N and water levels.

## Acknowledgements

The work was supported by the National Key R&D Program of China (2022YFD1900503, 2021YFD1900802), China Postdoctoral Science Foundation (2021 M703518), and the National Natural Science Foundation of China (51790534). We thank Prof. Xiaoling Su for providing the meteorological data of the Wuwei area under the future climate change.

## Author contributions

The study was designed by H.Z., S.K., G.V., M.G. and J.C.; H.Z., G.V., M.G., and J.C. contributed to the development of the integrative model; H.Z., G.V., and J.C. constructed the model and wrote the simulation code. H.Z. undertook the model testing and refinement. H.Z., S.K., M.G., and J.C. contributed to analysing the simulation results; H.Z. wrote the draft and all authors contributed to revising the paper.

## Data availability

The data that supports the findings were available in the paper and the Supplementary Materials published online.

## Conflicts of interest statement

The authors declare that they have no competing interests for this research.

## Supplementary data

Supplementary data is available at *Horticulture Research* online.

## Supplementary Material

Web_Material_uhad045
