# Supplementary material for: Integrated model simulates bigger, sweeter tomatoes under changing climate under reduced nitrogen and water input
Source: Hortic Res. 2023 Mar 13;10(5):uhad045. doi: 10.1093/hr/uhad045 (PMC10186270; doi:10.1093/hr/uhad045)
Supplement: Web_Material_uhad045 [file web_material_uhad045.docx]

**Supplementary Materials**

Method S1 Reduction functions of the N-CO_2_-Jarvis stomatal conductance model.

 (1)

$f(R^{'}_{n})=(k_{1}+0.55R^{'}_{n}/LAI)/(1+0.55R^{'}_{n}/LAI)$ (2)

$R^{'}_{n}=R_{n}\times[1-\exp(-k_{2}\times LAI)$ (3)

$f(T_{a})=1-k_{3}\cdot(25-T_{a})^{2}$ (4)

$f(VPD)=1-k_{4}\cdot VPD$ (5)

$f(\theta)=\left\{\begin{matrix} 1 \theta\geq\theta_{f} \\ \frac{\theta-\theta_{w}}{\theta_{f}-\theta_{w}} \theta_{w}\text{＜}\theta\text{＜}\theta_{f} \\ 0 \theta\leq\theta_{w} \end{matrix}\right.$ (6)

$f(N)=1.08\times\left[k_{n1}(\frac{N_{i}}{N_{1}}-1)(\frac{N_{i}}{N_{1}}-k_{n2})+1\right]$ (7)

$f(\mathrm{CO}_{2})=\frac{1}{1+b\left(\frac{CO_{2}}{CO_{2ref}}-1\right)}$ (8)

Net solar radiation, *R_n_* (MJ m^-2^ h^-1^), is calculated based on solar radiation together with location, time, and other meteorological information (Allen et al., 1998). *R_n_’* is the net solar radiation intercepted by tomato canopy. *LAI* is the leaf area index. *N_i_* is the soil nitrogen content, and *N_1_* is the baseline of the soil nitrogen content shown in Zhou et al., 2019. Variables and parameters are summarized in Table S1 and S2. Details of the calculation processes can be found in Li et al., 2019 and Zhou et al., 2019.

Allen, R.G., Pereira, L.S., Raes, D., Smith, M., 1998. Crop Evapotranspiration: Guidelines for Computing Crop Water Requirements. Irrigation and Drainage Paper No. 56. FAO, Rome, Italy, pp. 21–40.

Method S2 Method of linking the tomato fruit growth model with the fruit sugar model.

The soluble sugar content of fruits, necessary for the proper functioning of the tomato growth model, is provided by the TOM-SUGAR model, and the mass dynamics of the fruits, necessary for the functioning of the TOM-SUGAR model, is calculated by the tomato growth model. Thus, the tomato growth and TOM-SUGAR models were linked together.

The tomato fruit growth model could simulate tomato fruit fresh and dry weight dynamics by calculating the fruit water and carbon fluxes, where the fruit water flux included the water input from the xylem and phloem and the output of transpiration (Details in Liu et al., 2007). The fruit carbon flux was calculated as:

$\frac{ds}{dt}=U_{s}-R_{f}$ (1)

$U_{s}=U_{a}+U_{m}+U_{p}$ (2)

$U_{a}=\frac{s\times v_{m}\times Cp}{(K_{mfruit}+Cp)\times(1+\exp((\mathrm{t}-\mathrm{t}*)/\tau))}$ (3)

$U_{m}=(1-\sigma_{p})(Cp+C_{f})U_{p}/2$ (4)

$U_{p}=A_{p}p_{s}(Cp-C_{f})$ (5)

Where, *ds/dt* is the dynamics of fruit dry mass per unit time, g h^-1^; *U_s_* is the sucrose input from phloem to fruit per unit time, g h^-1^; *R_f_* is the fruit sucrose consumption by respiration, g h^-1^; *U_a_*, *U_m_* and *U_p_* are the sucrose input by active transport, mass flow and passive diffusion, respectively, g h^-1^. *K_mfruit_* is the Michaelis-Menten constant, 0.08 g cm^-3^; *p_s_* is the diffusion coefficient of sugar through membrane, 3.6*10^-5^ g cm^-2^h^-1^; *v_m_* is the capacity of active sugar transport across the membrane, g suc gDW^-1^ h^-1^; *t** (h) and *τ* are parameters driving the dynamics of the capacity of active sugar transport across the fruit during the fruit development (parameter details are shown in Liu et at., 2007 and Table S3); *Cp* is the sucrose concentration in phloem solution, *C_f_*, the fruit soluble sugar per unit water was calculated by TOM-SUGAR model as:

$C_{f}=\frac{C_{sol}}{c_{sol}\times w}$ (1)

$\frac{dC_{sol}}{dt}=U_{s}c_{suc}+k_{5}(t)C_{sta}-(k_{3}(t)+k_{5m}(t))C_{sol}-R_{f}c_{sol}$ (2)

$\frac{dC_{sta}}{dt}=k_{5m}(t)C_{sol}-k_{5}(t)C_{sta}$ (3)

$\frac{dC_{syn}}{dt}=k_{3}(t)C_{sol}$ (4)

$k_{3}(t)=\lambda((U_{s}-R_{f})/s)^{n}$ (5)

$k_{5}(t)=k_{5}$ (6)

$k_{5m}(t)=k_{5m0}/(1+e^{(t-u_{5m})/\tau_{5m}})$ (7)

Where, *w* is the fruit water content, g; *C_f_* is the fruit soluble sugar per unit water (g soluble sugar (g water)^-1^), *C_sol_*, *C_sta_* and *C_syn_* are and the carbon in form of soluble sugar, starch and other compounds in fruit, g C. *c_sol_* and *c_suc_* are the carbon amount in 1 g of soluble sugar and sucrose, the values are 0.4 g C (g soluble sugar)^-1^ and 0.421 g C (g sucrose)^-1^, respectively. *U_s_* and *R_f_* are sucrose input from phloem to fruit per unit time, g h^-1^, and fruit respiration, g h^-1^. t is the fruit development time, hours after anthesis, h. Parameters of tomato fruit sugar model (TOM-SUGAR) (Chen et al., 2020), *λ*, *n*, *k_5_*, *k_5m0_*, *u_5m_* and *τ_5m_*, are shown in Table S3.

Method S3 Experimental design and measurements in pot-grown tomato experimentations.

**Brief experimental design**

Experiments of three nitrogen application levels (no nitrogen application, N1; 0.1 g N kg^−1^ soil, N2; and 0.2 g N kg^−1^ soil, N3) with full irrigation (Wck) and deficit irrigation (receiving half the water amount of the full irrigation) were conducted in the summer of 2016 (**Experiment B**) and the winter of 2016–2017 (**Experiment C**) (Zhou et al., 2019; Zhou et al., 2020b). In 2015, only two nitrogen application levels (N2 and N3) were implemented (**Experiment A**). Details of the nitrogen and water applications for each experiment were summarized in the following Table MS3. For each treatment, 40 pots with one plant/pot were set for repetition.

Table MS3 Experimental design of different N and irrigation level in Experiments A, B, and C.

| Experiment | Treatment | N level | W level |
| --- | --- | --- | --- |
| B, C | N1Wck | N1 | Wck |
| A, B, C | N2Wck | N2 | Wck |
| B, C | N2DI | N2 | DI |
| A, B, C | N3Wck | N3 | Wck |

**Measurements**

**Meteorological factors**

Solar radiation (*R_s_*), air temperature (*T_a_*), and relative humidity (*RH*) were continuously recorded using a standard automatic weather station (Hobo, Onset Computer Corp., USA). All data were observed at 5 s intervals and recorded at 15 min intervals (Zhou et al., 2019).

**Soil water and mineral nitrogen content**

In experiments A, B, and C, soil water content in the root zone of the tomato plants was measured using 5TE soil moisture sensors (Decagon Devices Inc., USA). Data were collected every 15 min (Zhou et al., 2019).

Soil samples were taken from each treatment at intervals of 7 to 15 days during the growing season to obtain the NO_3_^−^-N (mg L^−1^) and NH_4_^+^-N (mg L^−1^) contents by a continuous flow analyzer (AutoAnalyzer3, Bran + Luebbe, SEAL Analytical GmbH, Germany) (Kamphake et al., 1967). The gravimetric soil water content was used to calculate the mineral nitrogen content (mg kg^−1^) of the soil.

**Organ growth**

The leaf area was measured at intervals of 5–7 days over the growing season (Zhou et al., 2019) in experiments A, B, and C. The fresh weights of the leaf, stem, and root were measured by sampling and dismembering the plant at intervals of 10–20 days over the growing season. Organ dry weights were obtained by drying the organ tissues to constant values by oven in experiments A, B, and C.

**Transpiration**

A heat balance-based Dynagage Flow32-1K system (Dynamax, Houston, Texas, USA) was used to monitor the dynamic change of tomato hourly sap flow during the winter experiments of 2016–2017 (Experiment C) (Zhou et al., 2019), which was used as a close approximation to plant transpiration, *Tr* (g h^-1^) (Baker and Bavel, 1987; Jiang et al., 2016).

**Leaf gas exchange**

Net photosynthetic rate and stomatal conductance of the tomato leaf were measured by a LI-6400 type photosynthesis system (Li-Cor 6400; Li-Cor, Lincoln, NE, USA). Daily variations in the photosynthetic parameters of each treatment were recorded at two-hour intervals from 8:00 to 18:00 in experiments A, B, and C (Zhou et al., 2020b).

In experiment B, mature leaves under different treatments were selected to obtain the A-Ci curves in sunny days at each growth stage, using portable photosynthesis systems (Li-Cor 6400; Li-Cor, Lincoln, NE, USA) with their 6 cm^2^ chamber fitted with the red-blue light source (Li-Cor 6400-02B LED) (Bloomfield et al., 2019).

**Stem and leaf water potential**

At each growth stage, three tomato plants were randomly selected for measuring the predawn and midday water potential of their leaf and stem at intervals of 10–15 d, using a pressure chamber (Model 1515D, PMS Instrument Company, USA). The potentials of leaf and stem were assumed to be equal at predawn, while leaves were wrapped with aluminum foil paper 4 hours in advance to avoid transpiration and measure the midday stem potential in experiments A, B, and C.

**Fruit weights and sugars**

Flowers on the second and third trusses were marked with their pollination time. Fruits were sampled according to the fruit age (days after anthesis) at intervals of 5–10 days. In each sampling, 3–5 fruits were picked for each treatment as repetition. The fruits were individually fresh weighted, and then an aliquot was used to obtain the fruit dry matter content and the fruit dry weight by oven drying the fruit tissue to constant weight at 80 ℃. The left part of the fruit was used to measure fruit soluble sugar and starch concentrations.

**References**

Kamphake LJ, Hannah SA, Cohen JM. 1967. Automated analysis for nitrate by hydrazine reduction. Water Resources 1, 205–216. 10.1016/0043-1354(67)90011-5

Baker JM and Bavel CHMV. 1987. Measurement of mass flow of water in the stems of herbaceous plants. Plant Cell and Environment 10, 777–782. 10.1111/1365-3040.ep11604765

Jiang XL, Kang SZ, Tong L, Li FS. 2016. Modification of evapotranspiration model based on effective resistance to estimate evapotranspiration of maize for seed production in an arid region of northwest China. Journal of Hydrology 538, 194–207. 10.1016/j.agwat.2016.05.022

Bloomfield KJ, Prentice IC, Cernusak LA, Eamus D, Medlyn BE, Rumman R, Wright IJ, Boer MM, Cale P, Cleverly J, Egerton JJG, Ellsworth DS, Evans BJ, Hayes LS, Hutchinson MF. 2019. The validity of optimal leaf traits modelled on environmental conditions. New Phytologist 221, 1409 – 1423. 10.1111/nph.15495

Figure S1 Dynamics of the hydraulic resistance from the soil to tomato stem (*Rp*) during the plant development stage (days after anthesis, DAA).

Figure S2 Dynamics of the maximal Rubisco carboxylation rate (*Vcmax*) and the maximal electron transport rate (*Jmax*) during the plant development stage under different water and N conditions. (DAA: days after anthesis)

Figure S3 Changes in air temperature (*T_a_*) and atmospheric CO_2_ concentration (*CO_2_*) in RCP4.5 for the Wuwei area from 2021–2100.

Figure S4 Calibration of structural carbon content in the leaf (*C_trs_*, A), non-structural carbon content in leaf (*C_ns_*, B), fruit soluble sugar content (*SS*, C), fruit starch content (*Sta*, D), and simulation of stomatal conductance (*g_sNCO2_*) and stem water potential (*ψ_stem_*) (E), leaf transpiration (*Tr*) and photosynthesis rate (*Pn*) (F), sucrose concentration in phloem solution (*Cp*, G), respiration of leaf, stem, root and fruit (H) along with days after anthesis (DAA).

Note: Datasets from N2Wck in Experiment A were used for calibration.

Figure S5 The simulated leaf structural carbon and non-structural carbon pool (*C_str_* and *C_ns_*) during 2021–2100.

For *C_str_*, the average value in the last 5 days of each growing season is taken as the final value for the season. For *C_ns_*, the mean of the whole growth season was used.

Figure S6 Simulated average leaf photosynthesis rate (*Pn_m*), stomatal conductance (*g_sNCO2__m*), transpiration accumulation (*Tc*), fruit net influx of carbon (*FruitC_net_*) and water (*FruitW_net_*) of each year during 2021–2100 and their relations with atmospheric CO_2_ concentration (*CO_2_*) and mean air temperature (*T_amean_*). *FruitC_net_* is the fruit carbon inflow from active transport, mass flow, and passive diffusion minus the outflow from respiration. *FruitW_net_* is the fruit water inflow from the xylem and phloem minus the outflow from fruit transpiration.

Table S1 Summary of the variables of the integrated model for tomato plant and fruit (TGFS).

| Variables | Definitions | Unit |
| --- | --- | --- |
| **N-CO_2_-Jarvis model** | | |
| *g_sNCO2_* | The stomatal conductance considering the effects of soil water, N and atmospheric CO_2_ concentration | mol H_2_O m^-2^ s^-1^ |
| *R_n_’* | The net solar radiation intercepted by tomato canopy | MJ m^-2^ h^-1^ |
| *LAI* | The leaf area index | m^2^ m^-2^ |
| *T_a_* | The air temperature | ℃ |
| *VPD* | The difference between the saturated vapor pressure and the actual vapor pressure | kPa |
| *θ* | The soil water content | cm^3^ cm^−3^ |
| *N* | The soil mineral nitrogen content | mg kg^−1^ |
| *CO_2_* | The atmospheric CO_2_ concentration | μmol mol^−1^ |
| *Pn* | The leaf gross photosynthesis rate | μmol CO_2_ m^-2^ s^-1^ |
|  | **Plant water module** |  |
| *Tr* | The transpiration of the big leaf | g h^-1^ |
| *LA* | The big leaf area | m^2^ |
| *ψ_stem_* | Tomato stem potential | MPa |
| *ψ_soil_* | Soil water potential | MPa |
| *Rp* | The resistance of water from soil to stem | MPa h g^-1^ |
| **Leaf carbon balance module** | | |
| *C_str_* | Leaf structural carbon | g C |
| *C_ns_* | Leaf non-structural carbon | g C |
| *Mresp_leaf_* | The carbon consumed by respiration in leaf per unit time | g C h^-1^ |
| *Loading_leaf_* | The amount of carbon that leaf unloaded into phloem per unit time | g C h^-1^ |
| *DW_leaf_* | Leaf dry weight | g |
| **Plant carbon allocation module** | | |
| *Uptake_stem_* | The carbon allocated to stem per unit time | g C h^-1^ |
| *Uptake_root_* | The carbon allocated to root per unit time | g C h^-1^ |
| *Uptake_fruit_* | The carbon allocated to fruit per unit time | g C h^-1^ |
| *C_stem_* | The carbon content in stem | g C |
| *Cp* | Sucrose concentration in phloem solution | mmol L^-1^ |
| *Mresp_stem_* | The carbon consumed by stem respiration per unit time | g C h^-1^ |
| *DW_stem_* | Stem dry weight | g |
| *C_root_* | The carbon content in root | g C |
| *Mresp_root_* | The carbon consumed by root respiration per unit time | g C h^-1^ |
| *DW_root_* | Root dry weight | g |
| **Fruit module** | | |
| *FW* | Individual fruit fresh weight | g |
| *DW* | Individual fruit dry weight | g |
| *SSc* | Fruit soluble sugar concentration | g/100g FW |
| *Stac* | Fruit starch concentration | g/100g FW |
| *SS* | Fruit soluble sugar content | g/100g DW |
| *Sta* | Fruit starch content | g/100g DW |

Table S2 Summary of the parameter values of stomatal conductance and plant carbon modules in the integrative model (TGFS).

| Parameters | | Definitions | Unit | | Values | Sources |
| --- | --- | --- | --- | --- | --- | --- |
| **g_sNCO2_ simulation** | | | | | | |
| *g_smax_* | Tomato maximum stomatal conductance | | | mol H_2_O m^-2^ s^-1^ | 0.576 | Measured |
| *θ_w_* | The wilting point of soil moisture | | | cm^3^ cm^−3^ | 0.14 | Zhou et al., 2019 |
| *θ_f_* | The field capacity of soil moisture | | | cm^3^ cm^−3^ | 0.28 | Zhou et al., 2019 |
| *CO_2ref_* | The reference CO_2_ concentration | | | μmol mol^−1^ | 330 | Li et al., 2019 |
| *b* | The parameter of the modified hyperbolic model considering the effect of CO_2_ on stomatal conductance | | | / | 0.663 | Li et al., 2019 |
| *k_1_* | A parameter describing the effect of solar radiation on stomatal conductance | | | / | 0.0327 | Calibrated |
| *k_2_* | The light extinction coefficient | | | / | 0.8 | Boulard et al., 1991; Qiu et al., 2013 |
| *k_3_* | A parameter describing the effect of T_a_ on stomatal conductance | | | / | 0.0016 | Calibrated |
| *k_4_* | A parameter describing the effect of VPD on stomatal conductance | | | / | 0.0025 | Calibrated |
| *k_n1_* | A parameter describing the effect of N on stomatal conductance | | | / | 0.021 | Calibrated |
| *k_n2_* | A parameter describing the effect of N on stomatal conductance | | | / | 0.76 | Calibrated |
| **Plant carbon module** | | | | | | |
| *K_ml_* | The maximal relative accumulation of structural carbo n mass in the leaf | | h^-1^ | | 0.013 | Zhu et al., 2019 |
| *SLAs* | The specific leaf area of structural carbon | | m^2^ (g C_str_)^-1^ | | 0.04 | Measured |
| *V_maxleaf_* | The maximum leaf carbon loading rate per unit leaf area. | | g C m^-2^ h^-1^ | | 3.80 | Zhu et al., 2019 |
| *K_mleaf_* | The Michaelis-Menten constant for leaf carbon loading | | g C (g FW)^-1^ | | 12.4 | Zhu et al., 2019 |
| *K_p→stem_* | The maximum rate coefficient of stem growth | | L (g C)^-1^ h^-1^ | | 1.44×10^-4^ | Calibrated |
| *A_p→stem_* | Attenuation coefficient of stem growth rate | | L (g C)^-1^ h^-1^ | | 1.36×10^-3^ | Calibrated |
| *B_p→stem_* | Time coefficient of stem growth rate attenuation | | h | | 720 | Observed |
| *K_p→root_* | Maximum rate coefficient of root growth | | L (g C)^-1^ h^-1^ | | 6.71×10^-5^ | Calibrated |
| *A_p→root_* | Attenuation coefficient of root growth rate | | L (g C)^-1^ h^-1^ | | 9.94×10^-4^ | Calibrated |
| *B_p→root_* | Time coefficient of root growth rate attenuation | | h | | 720 | Observed |
| *c_leaf_* | The carbon amount in 1 g of leaf dry mass | | g C (g DW)^-1^ | | 0.38 | Gary et al., 1998 |
| *c_stem_* | The carbon amount in 1 g of stem dry mass | | g C (g DW)^-1^ | | 0.36 | Gary et al., 1998 |
| *c_root_* | The carbon amount in 1 g of root dry mass | | g C (g DW)^-1^ | | 0.37 | Gary et al., 1998 |
| *q_mleaf_* | Maintenance respiration coefficient of leaf | | g sucrose (g DW)^-1^ h^-1^ | | 3.36×10^-4^ | Cieslak et al., 2011 |
| *q_mstem_* | Maintenance respiration coefficient of stem | | g sucrose (g DW)^-1^ h^-1^ | | 2.20×10^-4^ | Cieslak et al., 2011 |
| *q_mroot_* | Maintenance respiration coefficient of root | | g sucrose (g DW)^-1^ h^-1^ | | 5.96×10^-5^ | Cieslak et al., 2011 |
| *q_gleaf_* | Growth respiration coefficient of leaf | | g sucrose (g DW)^-1^ | | 0.2 | Gary et al., 1998 |
| *q_gstem_* | Growth respiration coefficient of stem | | g sucrose (g DW)^-1^ | | 0.1 | Gary et al., 1998 |
| *q_groot_* | Growth respiration coefficient of root | | g sucrose (g DW)^-1^ | | 0.1 | Gary et al., 1998 |
| *Q_10_* | A temperature sensitivity coefficient for maintenance respiration | | / | | 1.4 | Bertin and Heuvelink, 1993 |

Table S3 Summary of the parameter values of the fruit module in the integrative model (TGFS) including tomato fruit growth and fruit sugar model.

| Parameters | Definitions | Unit | Values | | Sources | |
| --- | --- | --- | --- | --- | --- | --- |
| **Tomato fruit growth model** | | | | | | |
| *v_m_* | The capacity of active sugar transport across the membrane | g sucrose (g DW)^-1^ h^-1^ | | 5.60×10^-2^ | | Calibrated |
| *t** | The parameter driving the dynamics of the capacity of active sugar transport across membrane during fruit development | h | | 28.51 | | Calibrated |
| *τ* | The parameter driving the dynamics of the capacity of active sugar transport across membrane during fruit development | / | | 851.23 | | Calibrated |
| *axp* | Ratio of vascular networks to fruit surface | / | | 2.50×10^-2^ | | Calibrated |
| *k_phi_* | A parameter describing the variation of cell wall extensibility. | h^-1^ | | 4.75×10^-3^ | | Calibrated |
| *τ_s_* | A constant coefficient | h^-2^ | | 1.53×10^-6^ | | Calibrated |
| *c_fuit_* | The carbon amount in 1 g of fruit dry mass | g C (g DW)^-1^ | | 0.44 | | Gary et al., 1998 |
| *q_mfruit_* | Maintenance respiration coefficient of fruit | g sucrose (g DW)^-1^ h^-1^ | | 0.00042 | | Thornley and Cannell, 2000 |
| *q_gfruit_* | Growth respiration coefficient of leaf | g sucrose (g DW)^-1^ | | 0.22 | | Gary et al., 1998 |
| *Fn* | Fruit number | / | | 10 | | Default |
| **Tomato fruit sugar model** | | | | | | |
| *λ* | A parameter describing soluble sugar conversion to structural substances | / | 2.23 | | Calibrated | |
| *n* | An index parameter describing soluble sugar conversion to structural substances | / | 1.10 | | Calibrated | |
| *k_5_* | A parameter describing starch conversion to soluble sugar | h^-1^ | 0.21 | | Calibrated | |
| *k_5m0_* | The initial rate of soluble sugar conversion to starch | h^-1^ | 0.55 | | Calibrated | |
| *u_5m_* | Time coefficient of conversion of soluble sugar to starch | h | 70.41 | | Calibrated | |
| *τ_5m_* | Rate coefficient of conversion of soluble sugar to starch | h | 288.65 | | Calibrated | |
